# Antimicrobial Resistance, Multilocus Sequence, and *spa* Typing of *Staphylococcus aureus* Isolated from Retail Raw Meat Products

**DOI:** 10.1155/2022/6035987

**Published:** 2022-09-14

**Authors:** Fatma Özdemir

**Affiliations:** Faculty of Arts and Sciences, Department of Biology, Bolu Abant Izzet Baysal University, 14030 Gölköy, Bolu, Turkey

## Abstract

With a high capacity to acquire antimicrobial resistance, *Staphylococcus aureus* is an important pathogen causing severe infections in animals and humans. A total of 50 *Staphylococcus aureus* isolates from retail ground beef, chicken meat, and fish were characterized by antimicrobial resistance profiling, staphylococcal protein A gene (*spa*) typing, and multilocus sequence typing (MLST). The broth microdilution test results showed that all isolates were resistant to penicillin and sulphamethoxazole but had varying resistance rates to tetracycline (24%), erythromycin (4%), gentamicin (2%), ciprofloxacin (2%), trimethoprim (2%), and chloramphenicol (0%). The *blaZ* and *sulI* genes were detected in 100% of the isolates followed by *grlA* (94%), *norA* (92%), *tetK* (80%), *chlA* (60%), *tetM* (26%), *aacA-aphD* (2%), *ermA* (2%), *fexA* (0%), and *dfrA* (0%). Moreover, 26% of the isolates were multidrug-resistant, with five or more resistance genes. The *spa* typing analysis revealed 22 *spa* types, with t091 (16%), t1677 (8%), and t14538 (8%) being the most common, and one new *spa* type, t19851, was uncovered. MLST identified seven sequence types (STs), with ST7 (40%), ST15 (20%), and ST199 (13%) being the most common, and two STs (ST7435 and ST7436) were newly identified. In this study, *S. aureus* isolated from raw meat showed multidrug resistance and different clones associated with human infections. As a result, foods of animal origin may act as potential vehicles for transmission of multidrug-resistant *S. aureus* isolates, and the dissemination of potentially pathogenic clonal types, posing a health risk to humans.

## 1. Introduction

*Staphylococcus aureus* is a commensal bacterium and colonizes the skin and mucous membranes of animals and humans. It causes various diseases in both animals and humans such as impetigo, skin infections, gastrointestinal tract infections, urinary tract infections, pneumonia, acute endocarditis, osteomyelitis, meningitis, enterocolitis, toxic shock syndrome, food poisoning, mastitis, and dermatitis [1]. Food contaminated with this pathogen may act as a vehicle of infection. The presence of *S. aureus* in raw foods such as meat and fish indicates poor personal hygiene [2]. *S. aureus* has often been isolated from a variety of foods of animal origin such as fish [3] and retail meat [4–6] as well as milk and milk products [7, 8].

Antimicrobial resistance is an increasing global threat to both human and animal health. The extended use or misuse of antimicrobials in human therapy, veterinary medicine, animal farming, and agricultural settings facilitates potential emergence and development of antimicrobial resistance. Foods of animal origin may be an important source for the transfer of antimicrobial-resistant *S. aureus* and antimicrobial resistance genes to humans [2, 9]. *S. aureus* has developed resistance against antimicrobials through mutation or horizontal genetic transfer of mobile genetic elements [10]. Methicillin-sensitive *S. aureus* (MSSA) generally evolves into methicillin-resistant *S. aureus* (MRSA) via the acquisition and insertion of staphylococcal chromosome cassette *mec* element which contains the gene *mecA*, a central genetic determinant of methicillin resistance in *S. aureus* and coagulase-negative staphylococci [11]. All MRSA strains harbor the *mecA* gene that encodes the penicillin-binding protein 2a (PBP2a). PBP2a exhibits low affinity for beta-lactam antimicrobials, which results in resistance to all beta-lactams [1, 10]. MRSA, which is a major cause of both community- and hospital-acquired infections, has received increasing attention due to its high pathogenicity and multidrug-resistant properties. The treatment of infections caused by MRSA is difficult with conventional antibiotics such as beta-lactams [2, 10]. Moreover, *S. aureus*, both MSSA and MRSA, has developed resistance to several antimicrobial classes including beta-lactams, macrolides, tetracyclines, aminoglycosides, amphenicols, fluoroquinolones, and sulfonamides which are prescribed in treatment of humans [10, 12]. These antimicrobials are also frequently used in food animals and aquaculture across the world to ensure abundant production of food [5, 7, 9, 13]. Antimicrobial-resistant MSSA and MRSA have been reported in retail meats [5, 6], ready-to-eat seafoods [13], and food animals [14].

Several molecular typing methods such as pulsed-field gel electrophoresis (PFGE), multilocus sequence typing (MLST), and staphylococcal protein A (*spa*) typing are available for epidemiological studies and differentiation of *S. aureus* isolates [8, 15, 16]. Among these methods, PFGE is still considered the gold standard for bacteria typing. MLST is a highly discriminatory method and characterizes the isolates using the sequences of the inner fragments of seven housekeeping genes. MLST data can be used to understand the evolutionary and population structure of *S. aureus* isolates that occur not only in a given region of a country but also allow comparison to clonal types from around the world [15, 17]. Another widely accepted method is *spa* typing which has become one of the most successful sequence-based typing approaches and has proven effective for distinguishing *S. aureus* from various sources as well as for nosocomial infection control [4, 17–19]. This method, based on sequence variation and the number of tandem repeats in the X region of the *spa* gene, displays excellent discriminatory power and has been described as a useful typing tool because of its ease of use, reproducibility, and standardized international nomenclature [16, 17, 20]. Previous studies showed that there was a strong correlation between *spa* typing and other typing methods such as MLST and PFGE [8, 16, 21].

Foods of animal origin are considered a potential source for the transmission of antimicrobial-resistant *S. aureus* strains to humans [2, 9]. To assess possible health risks, it is important to understand the pathogenic potential of *S. aureus* isolates. Therefore, the aim of this study was to investigate phenotypic and genotypic patterns of antimicrobial resistance and characterize *S. aureus* isolates using both *spa* and MLST typing in order to understand the molecular epidemiology of these isolates from retail raw meat products.

## 2. Materials and Methods

### 2.1. Bacterial Isolates

A total of 50 S*. aureus* isolates from 17 ground beef (cow's meat), 13 chicken meat (breast and leg parts), 20 fish (11 seawater fish (*Sparus aurata*), 8 freshwater fish (*Oncorhynchus mykiss*), and 1 seawater fish (*Dicentrarchus labrax*) samples were used in this study. *S. aureus* isolates were recovered from retail meat specimens collected from various public bazaars, supermarkets, and butchers in Bolu (Northwest Turkey). All isolates from different samples were previously identified using biochemical tests and a PCR for the species-specific fragment (Sa442) and thermonuclease gene (*nucA*) [1, 22–24]. Of the *S. aureus* isolates, 46 were MSSA and four were MRSA harboring the *mecA* gene as previously identified [25]. All isolates from retail meats were cultured in Brain Heart Infusion broth (BHI) (Merck, Germany) and incubated overnight at 37°C.

### 2.2. Phenotypic Detection of Antimicrobial Resistance

The minimum inhibitory concentrations (MICs) of eight antimicrobial agents were determined through the broth microdilution method in 96-well plates as per CLSI guidelines [26]. The following antimicrobial agents (HiMedia, Mumbai, India) were tested: penicillin G, gentamicin, chloramphenicol, tetracycline, erythromycin, ciprofloxacin, trimethoprim, and sulphamethoxazole. Plates were read with an ELISA reader (Thermo Electron Corporation, Vantaa, Finland). The MIC results were interpreted according to CLSI breakpoints [26]. The MIC breakpoints for the following antimicrobials (resistance breakpoints *μ*g/ml are in parentheses) were as follows: penicillin G (≥0.25), gentamicin (≥16), chloramphenicol (≥32), tetracycline (≥16), erythromycin (≥8), ciprofloxacin (≥4), trimethoprim (≥16), and sulphamethoxazole (≥512). *S. aureus* ATCC 29213 was included for quality control.

### 2.3. Genotypic Detection of Antimicrobial Resistance

Genomic DNA was extracted applying the cetyl trimethyl ammonium bromide (CTAB) method [27]. Implementing previously published primers and protocols, all *S. aureus* isolates were screened by PCR for antimicrobial resistance genes associated with beta-lactam resistance (*blaZ*), aminoglycoside resistance (*aacA-aphD*), florfenicol/chloramphenicol resistance (*chlA*, *fexA*), tetracycline resistance (*tetK*, *tetM*), macrolide resistance (*ermA*), quinolone resistance (*norA*, *grlA*), trimethoprim resistance (*dfrA*), and sulfonamide resistance (*sulI*) (Table S1). All PCR reactions were performed in a T100 thermal cycler (Bio-Rad, Hercules, USA). All amplified products analyzed by electrophoresis were visualized with a UV transilluminator (DNR Minilumi Bio-imaging Systems Ltd., Jerusalem, Israel). Positive controls were *S. aureus* ATCC 29213, *S. aureus* ATCC 25923, and *S. aureus* SA08 (*mecA*-positive from our collection).

### 2.4. Staphylococcal Protein A Gene (*spa*) Typing

The *spa* gene encoding protein A in the *S. aureus* isolates was amplified by PCR with the primers spa-1113f and spa-1514r (Table S1). The thermal cycling conditions were performed as described by Ridom Spa Server (https://www.ridom.de/doc/Ridom_spa_sequencing.pdf). All PCR products were purified with the HighPrep™ PCR Clean-up System (MAGBIO Genomics, Gaithersburg, MD, USA) and then sequenced using the BigDye Terminator v3.1 Cycle Sequencing kit (Applied Biosystems, Foster City, CA) in an ABI 3730 XL Genetic Analyzer (Applied Biosystems). *spa* typing was carried out through the Ridom Spa Server database (https://www.spaserver.ridom.de/) and Ridom SeqSphere + software version 7.6.1 (Ridom, Munster, Germany) for *spa* sequence analysis. The minimum spanning tree was constructed based on *spa* types for all isolates with the SeqSphere+ software. The *spa* types were clustered by BURP (based upon repeat pattern) analysis.

### 2.5. Multilocus Sequence Typing (MLST)

Thirteen multidrug-resistant (MDR) isolates out of 50 S*. aureus* isolates were chosen and characterized by the multilocus sequence typing (MLST) method to identify the epidemic lineages of MDR *S. aureus* which makes infection difficult to treat and poses a major threat to global health. In addition, two isolates representing a novel *spa* type (t19851) were also subjected to the MLST analysis. Seven housekeeping genes (*arcC*, *aroE*, *glpF*, *gmk*, *pta*, *tpi*, and *yqiL*) were amplified by PCR as described by Enright et al. [15] (Table S1). The allelic number of the genes and sequence type (ST) of each isolate were assigned according to the MLST database (https://pubmlst.org/organisms/staphylococcus-aureus). New MLST profiles were determined by sending the sequence trace files of the respective isolates to the database curator. Sequences were concatenated for each isolate and aligned using the ClustalW in MEGA 11 software (Version 11.0.10). A phylogenetic tree was constructed from the concatenated sequences by using the unweighted pair-group method with arithmetic mean (UPGMA).

### 2.6. Statistical Analysis

Statistical analyses were performed using SigmaPlot version 14.5 (Systat Software, Inc., San Jose, California, USA). One-way analysis of variance (ANOVA) with the Kruskal–Wallis test was applied to compare the difference in the phenotypic and genotypic resistance rates of the isolates from the various meat samples. The relationship between the phenotypic and genotypic resistance rates of the isolates was also determined using Pearson product moment correlation. Statistical significance was set at *p* ≤ 0.05.

## 3. Results

### 3.1. Antimicrobial Susceptibility Testing

**Figure 1:**
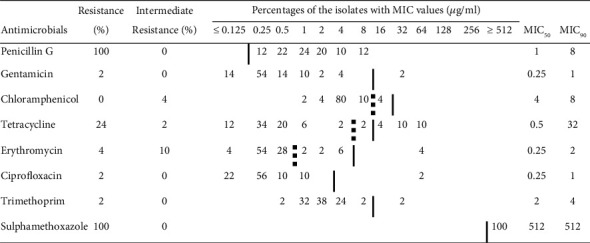
Minimum inhibitory concentrations (MICs) and antimicrobial resistance rates of the *S. aureus* isolates from retail raw meats. Bold vertical line indicates the MIC resistance breakpoint defined by CLSI [26]. Dashed bold vertical line indicates breakpoint for intermediate resistance

The MIC values and percentages of the isolates which were resistant or intermediate resistant to the antimicrobials included in the testing are summarized in Figure 1.

Antimicrobial resistance was observed to penicillin G and sulphamethoxazole (each 100%), followed by resistance to tetracycline (24%), erythromycin (4%), gentamicin (2%), ciprofloxacin (2%), and trimethoprim (2%). Conversely, resistance was not detected to chloramphenicol. Resistance to tetracycline was found in 41.2%, 30.8%, and 5% of the isolates from retail ground beef, chicken meat, and fish samples, respectively. However, no statistically significant difference was observed between the incidence of antimicrobial resistance and the isolates from different meat types (*p* =0.297). In total, 26% (13/50) of the isolates were determined to be resistant to at least three antimicrobials belonging to different classes and thus classified as multidrug-resistant (MDR). Of these, 100% of the MRSA isolated from chicken meat and 19.6% of the MSSA were MDR. Multidrug resistance among the MSSA isolates was 41.2% (7/17) in ground beef and 10% (2/20) in fish, respectively.

### 3.2. Antimicrobial Resistance Genes

**Figure 2:**
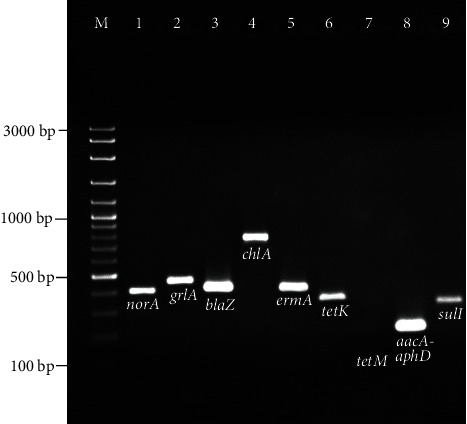
Agarose gel electrophoresis of PCR products of the representative *S. aureus* isolates carrying antimicrobial resistance genes. Lane M: 100 bp DNA ladder. Lanes 1-9: *norA* (406 bp), *grlA* (459 bp), *blaZ* (421 bp), *chlA* (768 bp), *ermA* (421 bp), *tetK* (360 bp), *tetM* (158 bp*), aacA-aphD* (227 bp), and *sulI* (331 bp) genes, respectively.

PCR amplicons of the resistance genes of the representative *S. aureus* isolates are shown in Figure 2.

Detection results of antimicrobial resistance genes in the *S. aureus* isolates from retail meat samples are presented in Table 1.

The *blaZ* and *sulI* genes were detected in all (100%) isolates which were also penicillin G- and sulphamethoxazole-resistant isolates based on the MIC test results. Other resistance genes detected were as follows: *grlA* (94%), *norA* (92%), *tetK* (80%), *chlA* (60%), *tetM* (26%), *aacA-aphD* (2%), *ermA* (2%), *fexA* (0%), and *dfrA* (0%). There was no significant difference between the prevalence of resistance genes and source of the isolates (*p* = 0.194). Phenotypic and genotypic resistance profiles of the *S. aureus* isolates among ground beef, chicken meat, and fish samples are shown in Table 2. Most of the isolates (≥80%) harbored the *tetK*, *grlA*, and *norA* genes. Twenty-seven of the 50 isolates had *tetK* alone. Thirteen isolates (26%) had both *tetK* and *tetM*. The *tetK* gene was detected in all tetracycline-resistant isolates. In contrast, 75.6% (28/37) of the tetracycline-sensitive isolates were positive for the *tetK* gene. The correlation analysis revealed that some of the resistance genes had a significant relationship with the corresponding phenotype such as *blaZ*, *aacA-aphD*, and *sulI* (*p* < 0.05). On the contrary, the presence of other genes (*chlA*, *fexA*, *tetK*, *tetM*, *ermA*, *grlA*, *norA*, and *dfrA*) was not significantly correlated with their phenotypic resistance (*p* > 0.05). Furthermore, the phenotypic resistance profiles of the isolates revealed that “PEN, SUL” was the most frequent (62%) pattern (Table 2).

### 3.3. *spa* Types

**Figure 3:**
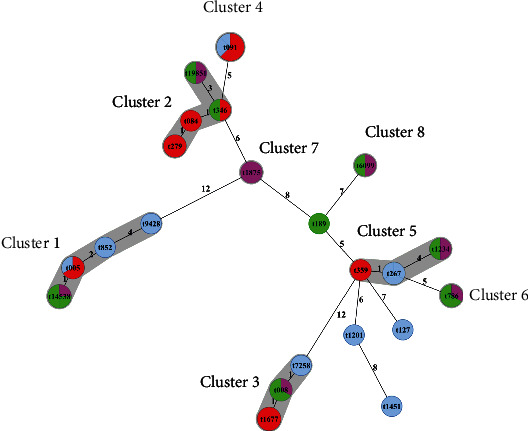
Minimum spanning tree of the 50 S*. aureus* isolates typed by *spa* typing. Each node represents a *spa* type, and the size of the circle corresponds to the number of isolates. The numbers on the edges between the nodes, which are calculated based on the BURP algorithm, indicate the distance between two *spa* types. Node colors refer to the source of the isolates allocated to the *spa* type (red, ground beef; blue, chicken meat; green, seawater fish; pink, freshwater fish).

In this study, the 50 *S. aureus* isolates were assigned to 22 different *spa* types with six to 15 repeats (Table 2). Two fish isolates, one freshwater and one seawater, were identified as novel *spa* types. These isolates were registered in the Ridom Spa Server database for the first time and designated as type t19851. The most common *spa* type present was t091 (16%; 8/50) followed by t14538, t1677 (both 8%; 4/50), t005, t008, t267, t279, t786 (each 6%; 3/50), and t1234, t1875, t19851, t346, t6099 (each 4%; 2/50). Each of the remaining 9 *spa* types accounted for 2% (1/50). The MSSA isolates had 18 different *spa* types. All four MRSA isolates belonged to different *spa* types, namely, t005, t7258, t852, and t9428. The *spa* types t005 and t091 were isolated both from chicken meat and ground beef. All the *spa* types identified in the freshwater fish isolates were also present in the seawater fish isolates except for one isolate that was assigned to t1875.  Figure 3 illustrates the minimum spanning tree that shows the distribution of the *spa* types by meat samples. Most of the *S. aureus* isolates (92%; *n* =46) were grouped into eight different clusters, and 8% (*n* = 4) of the isolates were evaluated as singletons (Figure 3). The discriminatory power of the *spa* typing method was 0.951 (95% confidence interval: 0.926-0.976).

### 3.4. MLST Analysis

**Figure 4:**
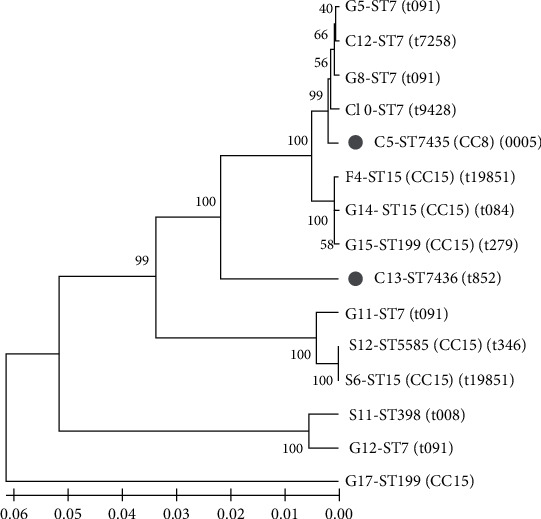
The unweighted pair-group method with arithmetic mean (UPGMA) phylogenetic tree of concatenated sequence of seven housekeeping genes. The scale bar indicates evolutionary distances in substitutions per site. Bootstrap *p* values (1000 replicates) are shown next to the branches. The sequence type (ST), clonal complex (CC), and *spa* type are given beside the isolate name. Isolate: G: ground beef; C: chicken meat; S: seawater fish; F: freshwater fish. Gray circles represent new ST types identified in this study.

A total of 15 *S. aureus* isolates, all 13 being MDR and two belonging to a novel *spa* type (t19851), were analyzed by the MLST method, and their allelic profiles as well as sequence types (STs) are given in Table 2. The UPGMA tree generated from MLST data illustrates the distribution of ST types among *S. aureus* isolated from raw meat products (Figure 4).

MLST analysis revealed that the tested 15 isolates had seven different ST types, with the most detected one being ST7 (40%, 6/15), followed by ST15 (20%, 3/15), and ST199 (13.3%, 2/15). The remaining four STs included ST398 (6.7%, 1/15), ST5585 (6.7%, 1/15), and two new STs: ST7435 (6.7%, 1/15) and ST7436 (6.7%, 1/15). The MLST data analyzed using the eBURST algorithm showed one group consisting of ST15, ST5585, and ST199 which ST15 (CC15) was the ancestral genotype, and four singletons, ST7, ST398, ST7435, and ST7436.

Of the 13 MDR isolates, seven MSSA ones from ground beef belonged to three ST types (ST7, ST15, and ST199) and one clonal complex, CC15 (*n* = 3, 42.9%), as well as two MSSA isolates from seawater fish one of which belonged to ST398 and the other to ST5585. The remaining four MDR isolates known as MRSA were from chicken meat half of which belonged to ST7 and the other half newly assigned to ST7435 (CC8) and ST7436. For these two novel ST isolates, details are available at PubMLST under the isolate IDs 37559 and 37560. Furthermore, two isolates (one from freshwater fish and one from seawater fish) exhibiting a new *spa* type, t19851, belonged to ST15 (CC15).

According to our results, some ST types corresponded to a single *spa* type, such as ST199 for t279, ST398 for t008, ST5585 for t346, ST7435 for t005, and ST7436 for t852. In contrast, one ST had multiple *spa* types such as ST7 (t091, t7258, t9428) and ST15 (t084 and newly identified t19851). There was no apparent link between the individual ST types and the types of the isolated sample (Figure 4).

## 4. Discussion

Excessive and imprudent use of antimicrobials for therapeutic purposes in veterinary and human medicine, or as growth promoters in animal husbandry, aquaculture, agriculture, and poultry are the main driving forces for the development and spread of resistant strains. The emergence and dissemination of multidrug-resistant *S. aureus*, and other various potentially pathogenic clones associated with human infections pose a public health risk [2, 5, 9].

Antimicrobial susceptibility of all the *S. aureus* (MSSA and MRSA) isolated from different meat products was tested using the broth microdilution method in this study. Overall, there was no significant difference in antimicrobial susceptibility rates among the isolates from different meat types (*p* = 0.297). All isolates displayed resistance to penicillin G and sulphamethoxazole (Figure 1). This result reflects higher use of these antimicrobials for treatment of diseases in cattle, poultry, and aquaculture farming [2, 9]. Moreover, similar to our findings, previous studies showed high levels of penicillin G resistance in the isolates of *S. aureus* from retail meats, food animals, and fishery products [3, 5, 7, 8, 14]. Penicillin G is commonly used to treat methicillin-sensitive *S. aureus* infections; however, most *S. aureus* strains are now resistant to penicillin G due to production of beta-lactamase, making other antimicrobials preferable for treatment of infections caused by *S. aureus* [10, 12]. In China, resistance to sulfafurazole, a sulfonamide, was detected in all (100%) MRSA and more than 80% of MSSA isolates from food animals [14], which was consistent with our results. In addition, Beshiru et al. [13] reported 73.6% of the *S. aureus* isolates from ready-to-eat seafood to be resistant to sulfonamides.

Tetracyclines as broad-spectrum antimicrobials are widely used for therapeutic purposes in humans and animals including cattle, poultry, and aquaculture as well as in animal feed as growth promoters [9, 12]. In the MIC results of this study, the percentages of tetracycline resistance in *S. aureus* from ground beef, chicken meat, and fish samples were 41.2%, 30.8%, and 5%, respectively. Similar results were obtained for tetracycline resistance in retail meat by Wang et al. [7] (42.8%) and Zehra et al. [6] (45.1%). Kim et al. [20] showed that tetracycline resistance level in chicken meat was 33.8%, which was parallel to our results. The incidence of our tetracycline-resistant isolates from ground beef was higher than in studies conducted in Georgia (25%) and Korea (2.1%) [5, 20], but lower than in a study done in the US (66.7%) [4]. In contrast to our result, 5%, related to fish samples, Vazquez-Sanchez et al. [3] found a higher proportion (86.7%) of tetracycline-resistant *S. aureus* isolates in fishery products.

Low resistance rates ranging from 2 to 4% were detected in the present study for erythromycin, ciprofloxacin, gentamicin, and trimethoprim which are highly effective antimicrobials for treating infections caused by Gram-positive and Gram-negative bacteria [2]. Erythromycin is used to treat less serious MSSA infections such as skin and soft tissue infections [12]. Several researchers have documented higher rates of resistance to erythromycin in *S. aureus* from retail meats varying from 10.4 to 52.1% [4, 6, 7, 20]. Resistance to ciprofloxacin was found between 1% and 2.7% in *S. aureus* from domestic and imported beef in a study performed in Korea [20], which was relatively closer to our results. A study in China showed that 17.4% of *S. aureus* isolates from raw meat had resistance to ciprofloxacin [7]. Contrary to the findings of this study, all *S. aureus* isolates (100%) were found to be resistant to ciprofloxacin in beef by Jackson et al. [5] in the USA and in fishery products by Vazquez-Sanchez et al. [3] in Spain. Gentamicin is clinically important for treating staphylococcal infections and is primarily used as a synergistic agent in treatment of staphylococcal endocarditis [12]. The frequency of gentamicin resistance (2%) among the isolates in this study was similar (2.1%) to that observed in domestic beef [20], but lower (15.1%) than in raw meats as reported by Wang et al. [7]. In addition, a higher level of resistance to gentamicin was reported by other researchers, particularly in MRSA [14, 28, 29]. However, several studies did not find gentamicin-resistant *S. aureus* isolates from beef, retail meats, and fishery products [3–5]. These differences in the resistance frequency among the isolates may be due to various factors such as the geographical regions studied and origins of the isolates [5, 14].

The overall prevalence of multidrug-resistant (MDR) *S. aureus* in this study was 26% (13/50), with MRSA 100% and MSSA 19.6%. Likewise, previous studies documented that the incidence of MDR in MRSA was higher than that in MSSA [14, 20, 28]. The prevalence of MDR isolates among ground beef, chicken meat, and fish samples was 41.2%, 30.8%, and 10%, respectively. The rate of MDR varies greatly across geographical regions in retail meats [5, 20]. In line with this, MDR *S. aureus* was present in 22.2% of retail meats in the United States [4], 45.6% of chicken meat in India [6], 62.4% of raw meat in China [7], and 100% of fishery products in Spain [3].

Antimicrobial resistance gene profiles were also detected by PCR for the *S. aureus* isolates from meat samples. According to the statistical analysis, no significant difference in the prevalence of resistance genes was present among the isolates from different meats (*p* = 0.194). All the MSSA and MRSA isolates carried both the *blaZ* and *sulI* genes encoding penicillin G and sulphamethoxazole resistance, respectively (Tables 1 and 2). The high prevalence of the *blaZ* gene (63.9-100%) was also demonstrated in previous studies from different countries [5, 14, 30]. A study by Beshiru et al. [13] showed that 92.3% of the *S. aureus* isolates from ready-to-eat seafood were positive for the *sulI* gene, which was in line with our results. The prevalence of tetracycline resistance genes *tetK* (80%) and *tetM* (26%) in this study was higher than that (20% for *tetK*, 6.7% for *tetM*) reported in retail food by Li et al. [31]. The rate of the *tetM* gene in our study (26%) was higher than that previously reported in *S. aureus* from various food samples (3.2%) [32]. The prevalence of *grlA* (94%) and *norA* (92%) genes associated with fluoroquinolone resistance in our study was considerably higher than that in earlier studies which ranged from 23.6% to 27.8% [30, 32]. The *aacA-aphD* gene encoding gentamicin resistance was detected only in one (2%) MRSA isolate from chicken meat. Previous studies indicated that the detection rates of this gene varied from 0 to 44.1% [5, 14, 28]. However, the *fexA* and *dfrA* genes were not found in this study, which was consistent with a previous study [28].

Comparison of the phenotypic and genotypic antimicrobial resistance results of *S. aureus* isolated from meat samples indicated that almost all phenotypically resistant isolates according to the MIC data harbored the related resistance genes (Table 2). Penicillin, sulphamethoxazole, and gentamicin were significantly correlated with their resistance genes (*p* < 0.05). However, no significant correlation was observed for other antimicrobials including ciprofloxacin, tetracycline, chloramphenicol, erythromycin, and trimethoprim (*p* > 0.05). The *grlA* and *norA* genes linked to fluoroquinolone resistance were found in more than 90% of the isolates whereas only one isolate was found to be resistant to ciprofloxacin (MIC ≥64 *μ*g/ml) (Figure 1 and Table 2). Not all positive isolates for the *tetK* gene in this study showed tetracycline resistance, similar to the findings of a previous study [5]. In addition, the *chlA* gene associated with chloramphenicol resistance was detected in 60% of the isolates, but we did not observe any isolates resistant to chloramphenicol (Figure 1 and Table 2). One seawater fish isolate was erythromycin-resistant (MIC ≥64 *μ*g/ml) whereas the isolate did not have the *ermA* gene, and likewise, a trimethoprim-resistant isolate (MIC ≥32 *μ*g/ml) did not carry the *dfrA* gene (Figure 1 and Table 2). These differences in the phenotype-genotype relationship may be due to an inactive or dysfunctional gene or the influence of other genetic and environmental factors [5, 10, 32].

Molecular characterization by *spa* typing revealed a wide genetic diversity with a total of 22 different *spa* types identified among the MSSA and MRSA isolates from ground beef, chicken meat, and fish (Table 2). In this study, the most prevalent *spa* type was t091 (16%) which was identified in the MSSA isolated from chicken meat and ground beef. This *spa* type has been documented in many European countries with 0.99% of global frequency, according to the Ridom Spa Server database (http://www.spaserver.ridom.de). Parallel to our findings, a study in China reported that *spa* type t091 was the most frequently observed one in the isolates from retail meats and meat products [21]. Interestingly, the most common *spa* type t091 was previously isolated from patients with skin and soft tissue infections in China [18]. Among our isolates, some identified *spa* types such as t005, t008, t084, t091, t127, t189, t267, t346, t359, and t786 were previously reported from Turkey [19, 33]. In the Netherlands, t091 and t084 as the most common *spa* types were also reported among clinical *S. aureus* isolates [34]. MSSA isolates from freshwater and seawater fish samples belonged to the *spa* types t008, t1234, t14538, t19851, t6099, and t786, of which t008 was the most prevalent clinical *spa* type in Europe [17]. Three fish isolates in this study were *spa* type t008 which was also found previously in chicken meat [4] and beef [5]. The *spa* type t189 identified in the seawater fish isolate in our study was also shown in the chicken and beef isolates in Korea [20]. Our results along with the findings of previous studies suggest that *spa* types vary among meat types. The *spa* types detected in the isolates of MRSA were t005, t7258, t852, and t9428. According to data on the Ridom Spa Server, the reported isolation frequency of MRSA *spa* types t005 and t852 was 0.70% and 0.13%, respectively. Having a very low global frequency (< 0.00%), the *spa* types t7258 and t9428 were reported in Canada and the United Kingdom, respectively, and for the first time in the current study in Turkey. The minimum spanning tree analysis based on *spa* typing revealed that the isolates were distributed among eight different clusters as shown in Figure 3. Cluster 4 comprised the *spa* type t091 which was the most prevalent in our isolates.

MLST data of all multidrug-resistant (MDR) and novel *spa* type isolates showed that ST7 was the most prevalent ST, followed by ST15, which was parallel to the results of a previous study on retail meats and meat products in China [21]. Another study from China found ST7 as the predominant type in *S. aureus* isolates associated with skin and soft tissue infections [18]. In the current study, ST398, ST5585, and ST15 were found in MSSA isolates from seawater fish (Table 2). A previous report showed that ST398 and ST15 were the predominant STs among sushi-associated MSSA isolates in China [31]. The most common clone in MSSA and MRSA isolates from retail meats and meat products in China was ST398 [21]. In the previous study from the United States, the presence of MRSA ST398 associated with pork was reported [4]. ST398 was also detected in MRSA and MSSA isolates associated with infections in humans [18].

Analysis of MLST data using the eBURST algorithm revealed one group and four singletons. ST15, ST199, and ST5585 were found in group 1 with type ST15 (CC15) as the ancestral type. ST15 was mainly associated with humans and reported as the most common lineage in both disease and carriage isolates in different studies [35, 36]. This clone was also frequently recorded from different origins in many countries, indicating geographical spread, according to the *S. aureus* MLST database.

To our knowledge, there is limited information on the MLST analysis of *S. aureus*, particularly MDR *S. aureus* from food samples including raw meats in Turkey. Furthermore, the identified STs, which were not reported in previous studies conducted in Turkey, were ST398, ST7, ST15, ST199, and ST5585 in the isolates from raw meats in the present study. Moreover, 13 MDR isolates from this study were grouped into seven STs and fell into clonal complexes CC8 and CC15 which were associated with MRSA and MSSA isolates, respectively. According to the *S. aureus* MLST database, CC8 isolates responsible for invasive infections recovered from blood were previously reported in Turkey. For the first time in Turkey, the MSSA isolates from ground beef and fish belonging to CC15 were identified in this study. In Europe, MRSA-associated with CC8 and MSSA-associated with CC15 were identified among bloodstream isolates [34].

## 5. Conclusions

This study demonstrated that all penicillin G- and sulphamethoxazole-resistant isolates carried the *blaZ* and *sulI* genes. All tetracycline-resistant isolates harbored the *tetK* gene. The majority of the isolates (≥80%) were positive for the *tetK*, *grlA*, and *norA* genes. In total, 26% of the isolates were multidrug-resistant (MDR) with five or more resistance genes. The rate of multidrug resistance was similar in the ground beef and chicken meat isolates, but lower in the fish. The molecular characterization by the *spa* and MLST typing revealed a high diversity among the *S. aureus* isolates and uncovered a new *spa* type (t19851) in the fish isolates as well as two unique STs (ST7435 and ST7436) in the chicken meat isolates. Overall, the findings highlighted the presence of MDR *S. aureus* and potentially pathogenic clones linked to human infections in retail meats. As a result, foods of animal origin may serve as a potential means for the transmission of these pathogens that entail a health risk to humans. Optimal use of antimicrobials should be ensured in animals and humans to control the growing hazard of antimicrobial resistance. Monitoring the antimicrobial resistance profiles and clonal types of *S. aureus* isolates is necessary for understanding epidemiological changes.

## Figures and Tables

**Table 1:** Frequency of resistance genes of the *S. aureus* isolates from retail raw meats.

	*β*-Lactam		Aminoglycoside		Chloramphenicol			Tetracycline			Macrolide		Quinolone			Trimethoprim		Sulphonamide	
	AR^a^	*blaZ*	AR^a^	*aacA*-*aphD*	AR^a^	*chlA*	*fexA*	AR^a^	*tetK*	*tetM*	AR^a^	*ermA*	AR^a^	*grlA*	*norA*	AR^a^	*dfrA*	AR^a^	*sulI*
Ground beef (*n* =17)																			
No. of isolates^b^	17	17	0	0	0	9	0	7	15	0	0	0	0	17	15	0	0	17	17
Prevalence (%)	100	100	0	0	0	52.9	0	41.2	88.2	0	0	0	0	100	88.2	0	0	100	100
Chicken meat (*n* =13)																			
No. of isolates^b^	13	13	1	1	0	13	0	4	13	12	1	1	1	10	11	1	0	13	13
Prevalence (%)	100	100	7.7	7.7	0	100	0	30.8	100	92.3	7.7	7.7	7.7	76.9	84.6	7.7	0	100	100
Seawater fish (*n* =12)																			
No. of isolates^b^	12	12	0	0	0	3	0	1	9	1	1	0	0	12	12	0	0	12	12
Prevalence (%)	100	100	0	0	0	25	0	8.3	75	8.3	8.3	0	0	100	100	0	0	100	100
Freshwater fish (*n* =8)																			
No. of isolates^b^	8	8	0	0	0	5	0	0	3	0	0	0	0	8	8	0	0	8	8
Prevalence (%)	100	100	0	0	0	62.5	0	0	37.5	0	0	0	0	100	100	0	0	100	100

^a^AR, the resistant isolates detected by the broth microdilution method. ^b^Number of the isolates positive for resistance phenotypes and genes encoding resistance to antimicrobials.

**Table 2:** Antimicrobial resistance, *spa* typing, and MLST analysis of *S. aureus* isolates from retail raw meat products.

SI no	Isolate^a^	Methicillin resistance^b^	Phenotypic resistance profile^c^	Genotypic resistance profile	*spa* typing		MLST^f^	
					*spa* type	Repeats	Allelic profile	ST (CC)
1	G1	MSSA	PEN, SUL	*blaZ*, *norA*, *grlA*, *sulI*	t005	26-23-13-23-31-05-17-25-17-25-16-28		
2	G2	MSSA	PEN, SUL	*blaZ*, *tetK*, *norA*, *grlA*, *sulI*	t279	07-23-12-34-34-34-12-12-23-02-12-23		
3	G3	MSSA	PEN, SUL	*blaZ*, *norA*, *grlA*, *sulI*	t1677	11-19-12-12-17-34-24-34-22-25		
4	G4	MSSA	PEN, SUL	*blaZ*, *tetK*, *norA*, *grlA*, *sulI*	t1677	11-19-12-12-17-34-24-34-22-25		
5	G5	MSSA	PEN, TET, SUL	*blaZ*, *chlA*, *tetK*, *norA*, *grlA*, *sulI*	t091	07-23-21-17-34-12-23-02-12-23	5-4-1-4-4-6-3	7
6	G6	MSSA	PEN, SUL	*blaZ*, *chlA*, *tetK*, *norA*, *grlA*, *sulI*	t1677	11-19-12-12-17-34-24-34-22-25		
7	G7	MSSA	PEN, SUL	*blaZ*, *tetK*, *norA*, *grlA*, *sulI*	t359	07-23-12-21-17-34-34-33-34		
8	G8	MSSA	PEN, TET, SUL	*blaZ*, *chlA*, *tetK*, *norA*, *grlA*, *sulI*	t091	07-23-21-17-34-12-23-02-12-23	5-4-1-4-4-6-3	7
9	G9	MSSA	PEN, SUL	*blaZ*, *tetK*, *grlA*, *sulI*	t1677	11-19-12-12-17-34-24-34-22-25		
10	G10	MSSA	PEN, SUL	*blaZ*, *tetK*, *norA*, *grlA*, *sulI*	t005	26-23-13-23-31-05-17-25-17-25-16-28		
11	G11	MSSA	PEN, TET, SUL	*blaZ*, *chlA*, *tetK*, *norA*, *grlA*, *sulI*	t091	07-23-21-17-34-12-23-02-12-23	5-4-1-4-4-6-3	7
12	G12	MSSA	PEN, TET, SUL	*blaZ*, *tetK*, *norA*, *grlA*, *sulI*	t091	07-23-21-17-34-12-23-02-12-23	5-4-1-4-4-6-3	7
13	G13	MSSA	PEN, SUL	*blaZ*, *chlA*, *tetK*, *norA*, *grlA*, *sulI*	t346	07-23-12-34-12-12-23-02-12-23		
14	G14	MSSA	PEN, TET, SUL	*blaZ*, *chlA*, *tetK*, *norA*, *grlA*, *sulI*	t084	07-23-12-34-34-12-12-23-02-12-23	13-13-1-1-12-11-13	15 (15)
15	G15	MSSA	PEN, TET, SUL	*blaZ*, *chlA*, *tetK*, *norA*, *grlA*, *sulI*	t279	07-23-12-34-34-34-12-12-23-02-12-23	13-13-1-1-12-1-13	199 (15)
16	G16	MSSA	PEN, SUL	*blaZ*, *chlA*, *tetK*, *grlA*, *sulI*	t091	07-23-21-17-34-12-23-02-12-23		
17	G17	MSSA	PEN, TET, SUL	*blaZ*, *chlA*, *tetK*, *norA*, *grlA*, *sulI*	t279	07-23-12-34-34-34-12-12-23-02-12-23	13-13-1-1-12-1-13	199 (15)
18	C1	MSSA	PEN, SUL	*blaZ*, *chlA*, *tetK*, *tetM*, *sulI*	t1451	08-16-02-25-34-25		
19	C2	MSSA	PEN, SUL	*blaZ*, *chlA*, *tetK*, *tetM*, *norA*, *grlA*, *sulI*	t127	07-23-21-16-34-33-13		
20	C3	MSSA	PEN, SUL	*blaZ*, *chlA*, *tetK*, *tetM*, *norA*, *grlA*, *sulI*	t1201	07-16-34-34-33-34		
21	C4	MSSA	PEN, SUL	*blaZ*, *chlA*, *tetK*, *tetM*, *norA*, *grlA*, *sulI*	t091	07-23-21-17-34-12-23-02-12-23		
22	C5	MRSA	PEN, GEN, CHL^d^, TET, ERY, CIP, TMP, SUL	*blaZ*, *aacA-aphD*, *chlA*, *tetK*, *tetM*, *ermA*, *norA*, *grlA*, *sulI*	t005	26-23-13-23-31-05-17-25-17-25-16-28	3-31-1-1-4-4-3	**7435 (8**)
23	C6	MSSA	PEN, SUL	*blaZ*, *chlA*, *tetK*, *tetM*, *norA*, *grlA*, *sulI*	t267	07-23-12-21-17-34-34-34-33-34		
24	C7	MSSA	PEN, SUL	*blaZ*, *chlA*, *tetK*, *tetM*, *norA*, *grlA*, *sulI*	t091	07-23-21-17-34-12-23-02-12-23		
25	C8	MSSA	PEN, SUL	*blaZ*, *chlA*, *tetK*, *norA*, *grlA*, *sulI*	t267	07-23-12-21-17-34-34-34-33-34		
26	C9	MSSA	PEN, SUL	*blaZ*, *chlA*, *tetK*, *tetM*, *norA*, *grlA*, *sulI*	t091	07-23-21-17-34-12-23-02-12-23		
27	C10	MRSA	PEN, TET, SUL	*blaZ*, *chlA*, *tetK*, *tetM*, *sulI*	t9428	07-23-13-23-31-05-17-25-17-25	5-4-1-4-4-6-3	7
28	C11	MSSA	PEN, SUL	*blaZ*, *chlA*, *tetK*, *tetM*, *norA*, *grlA*, *sulI*	t267	07-23-12-21-17-34-34-34-33-34		
29	C12	MRSA	PEN, TET, SUL	*blaZ*, *chlA*, *tetK*, *tetM*, *norA*, *grlA*, *sulI*	t7258	15-19-12-21-17-34-24-34-22-25	5-4-1-4-4-6-3	7
30	C13	MRSA	PEN, TET, ERY^d^, SUL	*blaZ*, *chlA*, *tetK*, *tetM*, *norA*, *sulI*	t852	07-23-13-23-31-05-17-25-17-25-16-28	13-4-1-4-4-287-13	**7436** ^g^
31	S1	MSSA	PEN, SUL	*blaZ*, *tetK*, *norA*, *grlA*, *sulI*	t1234	07-23-12-12-34-34-34-33-34		
32	S2	MSSA	PEN, SUL	*blaZ*, *tetK*, *norA*, *grlA*, *sulI*	t14538	26-23-13-23-31-05-23-31-05-17-25-17-25-16-28		
33	S3	MSSA	PEN, TET^d^ SUL	*blaZ*, *norA*, *grlA*, *sulI*	t189	07-23-12-21-17-34		
34	S4	MSSA	PEN, SUL	*blaZ*, *tetK*, *norA*, *grlA*, *sulI*	t14538	26-23-13-23-31-05-23-31-05-17-25-17-25-16-28		
35	S5	MSSA	PEN, ERY^d^, SUL	*blaZ*, *norA*, *grlA*, *sulI*	t14538	26-23-13-23-31-05-23-31-05-17-25-17-25-16-28		
36	S6	MSSA	PEN, SUL	*blaZ*, *tetK*, *norA*, *grlA*, *sulI*	**t19851** ^**e**^	07-23-12-12-34-12-12-23-20-12-23	13-13-1-1-12-11-13	15 (15)
37	S7	MSSA	PEN, SUL	*blaZ*, *chlA*, *tetK*, *norA*, *grlA*, *sulI*	t6099	07-23-12-21-17-34-33-16-16-16-23		
38	S8	MSSA	PEN, CHL^d^, SUL	*blaZ*, *tetK*, *norA*, *grlA*, *sulI*	t008	11-19-12-21-17-34-24-34-22-25		
39	S9	MSSA	PEN, SUL	*blaZ*, *tetK*, *norA*, *grlA*, *sulI*	t786	07-12-21-17-13-34-34-33-34		
40	S10	MSSA	PEN, SUL	*blaZ*, *chlA*, *norA*, *grlA*, *sulI*	t786	07-12-21-17-13-34-34-33-34		
41	S11	MSSA	PEN, ERY, SUL	*blaZ*, *tetK*, *norA*, *grlA*, *sulI*	t008	11-19-12-21-17-34-24-34-22-25	3-35-19-2-20-26-39	398
42	S12	MSSA	PEN, TET, SUL	*blaZ*, *chlA*, *tetK*, *tetM*, *norA*, *grlA*, *sulI*	t346	07-23-12-34-12-12-23-02-12-23	13-13-1-444-12-11-13	5585 (15)
43	F1	MSSA	PEN, SUL	*blaZ*, *chlA*, *norA*, *grlA*, *sulI*	t1234	07-23-12-12-34-34-34-33-34		
44	F2	MSSA	PEN, SUL	*blaZ*, *chlA*, *norA*, *grlA*, *sulI*	t6099	07-23-12-21-17-34-33-16-16-16-23		
45	F3	MSSA	PEN, SUL	*blaZ*, *chlA*, *tetK*, *norA*, *grlA*, *sulI*	t14538	26-23-13-23-31-05-23-31-05-17-25-17-25-16-28		
46	F4	MSSA	PEN, ERY^d^, SUL	*blaZ*, *norA*, *grlA*, *sulI*	**t19851** ^**e**^	07-23-12-12-34-12-12-23-20-12-23	13-13-1-1-12-11-13	15 (15)
47	F5	MSSA	PEN, ERY^d^, SUL	*blaZ*, *chlA*, *norA*, *grlA*, *sulI*	t1875	07-23-12-34-12-23		
48	F6	MSSA	PEN, SUL	*blaZ*, *tetK*, *norA*, *grlA*, *sulI*	t008	11-19-12-21-17-34-24-34-22-25		
49	F7	MSSA	PEN, SUL	*blaZ*, *chlA*, *norA*, *grlA*, *sulI*	t786	07-12-21-17-13-34-34-33-34		
50	F8	MSSA	PEN, ERY^d^, SUL	*blaZ*, *tetK*, *norA*, *grlA*, *sulI*	t1875	07-23-12-34-12-23		

^a^G: ground beef; C: chicken meat; S: seawater fish; F: freshwater fish. ^b^MSSA: methicillin-sensitive *S. aureus*; MRSA: methicillin-resistant *S. aureus* harboring the *mecA* gene. ^c^PEN: penicillin G; SUL: sulphamethoxazole; ERY: erythromycin; TET: tetracycline; CHL: chloramphenicol; GEN: gentamicin; CIP: ciprofloxacin; TMP: trimethoprim. ^d^Intermediate resistance to the indicated antimicrobial agent according to CLSI standards. ^e^New *spa* types are shown in bold font. ^f^Multilocus sequence typing (MLST) was conducted for 13 multidrug-resistant isolates (three or more antimicrobial classes) and for two isolates belonging to a new *spa* type. ST: sequence type; CC: clonal complex. ^g^New STs types are identified by bold type.

## Data Availability

The datasets used and/or analyzed in the current study are available from the corresponding author on reasonable request.

## Supplementary Materials

Supplementary Materials Table S1. Primer sequences with their corresponding PCR conditions and product sizes for each of the genes analyzed [37–43].
