# Correction to “Antimicrobial Resistance, Multilocus Sequence, and Spa Typing of *Staphylococcus aureus* Isolated From Retail Raw Meat Products”

**DOI:** 10.1155/bmri/9781902

**Published:** 2025-10-10

F. Özdemir, “Antimicrobial Resistance, Multilocus Sequence, and *Spa* Typing of *Staphylococcus aureus* Isolated From Retail Raw Meat Products,” *BioMed Research International* 2022 (2022): 6035987, https://doi.org/10.1155/2022/6035987

This article contains an error in Figure 2, in which the band for *tetM* was omitted during the production process. The correct Figure 2 is shown in the following:

**Figure 2:**
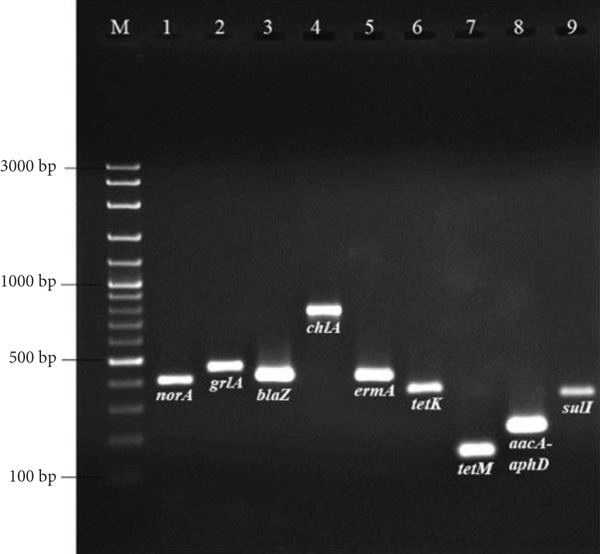
Agarose gel electrophoresis of PCR products of the representative *S. aureus* isolates carrying antimicrobial resistance genes. Lane M: 100 bp DNA ladder. Lanes 1–9: norA (406 bp), grlA (459 bp), blaZ (421 bp), chlA (768 bp), ermA (421 bp), tetK (360 bp), tetM (158 bp), aacA‐aphD (227 bp), and sulI (331 bp) genes, respectively.

We apologize for this error.

