# Supplementary material for: Antimicrobial Resistance, Multilocus Sequence, and spa Typing of Staphylococcus aureus Isolated from Retail Raw Meat Products
Source: Biomed Res Int. 2022 Sep 14;2022:6035987. doi: 10.1155/2022/6035987 (PMC11401655; doi:10.1155/2022/6035987)
Supplement: Supplementary Materials — Table S1. Primer sequences with their corresponding PCR conditions and product sizes for each of the genes analyzed [37–43]. [file 6035987.f1.docx]

**Table S1.** Primer sequences with their corresponding PCR conditions and product sizes for each of the genes analyzed.

| Target gene | Primer sequences (5′-3′) | Product size (bp) | PCR condition | Reference |
| --- | --- | --- | --- | --- |
| **Antimicrobial resistance genes** | |  |  |  |
| *blaZ* | F: CAA AGA TGA TAT AGT TGC TTA TTC TCC  R: TGC TTG ACC ACT TTT ATC AGC | 421 | 94 °C for 5 min, 35× (94 °C for 30 s, 55 °C for 30 s, 72 °C for 30 s), 72 °C for 7 min | [29] |
| *aacA-aphD* | F: TAA TCC AAG AGC AAT AAG GGC  R: GCC ACA CTA TCA TAA CCA CTA | 227 | 94 °C for 3 min, 30× (94 °C for 30 s, 58 °C for 30 s, 72 °C for 60 s), 72 °C for 4 min | [37] |
| *chlA* | F: CCT GCT AAC AAT AGA CCT GA  B: CGC TTT AAC ATT TGC GAT AT | 768 | 94 °C for 5 min, 35× (95 °C for 40 s, 59 °C for 55 s, 72 °C for 40 s), 72 °C for 10 min | [38] |
| *fexA* | F: GTA CTT GTA GGT GCA ATT ACG GCT GA  R: CGC ATC TGA GTA GGA CAT AGC GTC | 1272 | 94 °C for 1 min, 34× (94 °C for 60 s, 57 °C for 2 min, 72 °C for 3 min), 72 °C for 7 min | [39] |
| *tetK* | F: GTA GCG ACA ATA GGT AAT AGT  R: GTA GTG ACA ATA AAC CTC CTA | 360 | 94 °C for 5 min, 30× (94 °C for 60 s, 58 °C for 70 s, 72 °C for 60 s), 72 °C for 10 min | [37] |
| *tetM* | F: AGT GGA GCG ATT ACA GAA  R: CAT ATG TCC TGG CGT GTC TA | 158 |  |  |
| *ermA* | F: GTT CAA GAA CAA TCA ATA CAG AG  R: GGA TCA GGA AAA GGA CAT TTT AC | 421 | 94 °C for 3 min, 30× (94 °C for 30 s, 52 °C for 30 s, 72 °C for 60 s), 72 °C for 4 min | [40] |
| *norA* | F: TAT CGG TTT AGT ATT ACC AGT C  R: AAC TTC TGC CAT AAA TCC AC | 406 | 94 °C for 5 min, 30× (94 °C for 60 s, 60 °C for 30 s, 72 °C for 30 s), 72 °C for 10 min | [38] |
| *grlA* | F: ACT TGA AGA TGT TTT AGG TGA T  R: TTA GGA AAT CTT GAT GGC AA | 459 | 94 °C for 10 min, 25× (94 °C for 20 s, 52 °C for 20 s, 72 °C for 50 s), 72 °C for 5 min | [41] |
| *dfrA* | F: CTC ACG ATA AAC AAA GAG TCA  R: CAA TCA TTG CTT CGT ATA ACG | 201 | 94 °C for 2 min, 30× (94 °C for 60 s, 50 °C for 60 s, 72 °C for 60 s), 72 °C for 5 min | [42] |
| *sulI* | F: TCA CCG AGG ACT CCT TCT TC  R: CAG TCC GCC TCA GCA ATA TC | 331 | 95 °C for 10 min, 30× (95 °C for 30 s, 65 °C for 60 s, 72 °C for 60 s), 72 °C for 7 min | [43] |

**Table S1 (continue)**

| Target gene | Primer sequences (5′-3′) | Product size (bp) | PCR condition | Reference |
| --- | --- | --- | --- | --- |
| **Staphylococcal protein A (*spa*) typing** | |  |  |  |
| *spa* | spa-1113f: TAA AGA CGA TCC TTC GGT GAG C  spa-1514r: CAG CAG TAG TGC CGT TTG CTT | Variable | 80 °C for 5 min, 35× (94 °C for 45 s, 60 °C for 45 s, 72 °C for 90 s), 72°C for 10 min | [16] |
| **Multilocus sequence typing (MLST)** | |  |  |  |
| *arcC* | arc F: TTG ATT CAC CAG CGC GTA TTG TC  arc R: AGG TAT CTG CTT CAA TCA GCG | Variable | 95 °C for 5 min, 30× (95 °C for 60 s, 55 °C for 60 s, 72 °C for 30 s), 72°C for 5 min | [15] |
| *aroE* | aro F: ATC GGA AAT CCT ATT TCA CAT TC  aro R: GGT GTT GTA TTA ATA ACG ATA TC |  |  |  |
| *glpF* | glp F: CTA GGA ACT GCA ATC TTA ATC C  glp R: TGG TAA AAT CGC ATG TCC AAT TC |  |  |  |
| *gmk* | gmk F: ATC GTT TTA TCG GGA CCA TC  gmk R: TCA TTA ACT ACA ACG TAA TCG TA |  |  |  |
| *pta* | pta F: GTT AAA ATC GTA TTA CCT GAA GG  pta R: GAC CCT TTT GTT GAA AAG CTT AA |  |  |  |
| *tpi* | tpi F: TCG TTC ATT CTG AAC GTC GTG AA  tpi R: TTT GCA CCT TCT AAC AAT TGT AC |  |  |  |
| *yqiL* | yqi F: CAG CAT ACA GGA CAC CTA TTG GC  yqi R: CGT TGA GGA ATC GAT ACT GGA AC |  |  |  |
